# MAGIC-web: a platform for untargeted and targeted N-linked glycoprotein identification

**DOI:** 10.1093/nar/gkw254

**Published:** 2016-04-15

**Authors:** T. Mamie Lih, Wai-Kok Choong, Chen-Chun Chen, Cheng-Wei Cheng, Hsin-Nan Lin, Ching-Tai Chen, Hui-Yin Chang, Wen-Lian Hsu, Ting-Yi Sung

**Affiliations:** 1 Bioinformatics Program, Taiwan International Graduate Program, Academia Sinica, Taipei 11529, Taiwan; 2 Institute of Information Science, Academia Sinica, Taipei 11529, Taiwan; 3 Institute of Biomedical Informatics, National Yang-Ming University, Taipei 11221, Taiwan; 4 Genomics Research Center, Academia Sinica, Taipei 11529, Taiwan; 5 Department of Chemistry, National Taiwan University, Taipei 10617, Taiwan

## Abstract

MAGIC-web is the first web server, to the best of our knowledge, that performs both untargeted and targeted analyses of mass spectrometry-based glycoproteomics data for site-specific N-linked glycoprotein identification. The first two modules, MAGIC and MAGIC+, are designed for untargeted and targeted analysis, respectively. MAGIC is implemented with our previously proposed novel Y1-ion pattern matching method, which adequately detects Y1- and Y0-ion without prior information of proteins and glycans, and then generates *in silico* MS^2^ spectra that serve as input to a database search engine (e.g. Mascot) to search against a large-scale protein sequence database. On top of that, the newly implemented MAGIC+ allows users to determine glycopeptide sequences using their own protein sequence file. The third module, Reports Integrator, provides the service of combining protein identification results from Mascot and glycan-related information from MAGIC-web to generate a complete site-specific protein-glycan summary report. The last module, Glycan Search, is designed for the users who are interested in finding possible glycan structures with specific numbers and types of monosaccharides. The results from MAGIC, MAGIC+ and Reports Integrator can be downloaded via provided links whereas the annotated spectra and glycan structures can be visualized in the browser. MAGIC-web is accessible from http://ms.iis.sinica.edu.tw/MAGIC-web/index.html.

## INTRODUCTION

Protein glycosylation involves numerous biological relevant events, such as cell adhesion (1,2), protein folding (3) and immune responses (4). It is a commonest and highly complex post-translation modification that alterations in glycosylation are observed in association with cancers (5,6), inherited disorders (7,8) and other diseases (9). However, in view of the diversity in glycan structures and site-specific heterogeneity of glycan occupancy, glycoproteomic analysis remains technically challenging. Currently, mass spectrometry (MS) has been widely adopted for studying glycoproteomics due to its high speed and high sensitivity (10,11). Applying tandem MS to analyse intact glycopeptides retaining site-specific glycans has the advantage of possible inference of glycosylation sites and glycan compositions. Nonetheless, using the conventional collision-induced dissociation (CID) technique in MS, B- and Y-ions (resulting from sequential neutral losses of monosaccharides at the terminal positions) often dominate in the acquired tandem mass spectrometry (MS^2^) data, causing a great difficulty to identify the underlying peptides since b- and y-ions of the peptides are relatively few and low–abundance. To tackle the computational challenge of analysing intact glycopeptide MS^2^ spectra, currently only few web servers are available that include GlycoPep DB (12), GlycoPep ID (13), GlycoPep Grader (14), GlycoPep Detector (15) and GlycoMaster DB (16). However, all of them are limited to targeted analysis and require users to input protein/peptide sequences or peptide masses and/or glycan information due to data intractability. To the best of our knowledge, there is yet a web server that could perform the targeted analysis while supporting the untargeted analysis as well. Therefore, we have developed MAGIC-web to provide automated site-specific N-linked glycosylation analysis from acquired beam-type CID MS^2^ data for both untargeted and targeted glycoprotein identification. To be specific, MAGIC-web contains the following four modules: MAGIC (17), MAGIC+, Reports Integrator and Glycan Search. MAGIC (Mass spectrometry-based Automated Glycopeptide IdentifiCation platform) is previously published as a stand-alone program executable on the Windows platform (17). It can identify intact N-glycosylated peptides from a public protein database without requiring any prior information of proteins or glycans, and its performance has been demonstrated in the manuscript. MAGIC aims to support untargeted glycopeptide analysis while the newly implemented MAGIC+ is designed to perform targeted glycopeptide analysis that allows users to upload their own protein sequence file to find glycopeptides in their data. The search results from Mascot can be integrated with the results from MAGIC-web via Reports Integrator to generate a complete protein/peptide-glycan summary report. Independent of the above three modules, the fourth module, Glycan Search, allows users to find various glycans from a large glycan database stored in the web server, regardless the types of glycosylation.

MAGIC-web is accessible at http://ms.iis.sinica.edu.tw/MAGIC-web/index.html with standard web browsers such as Chrome, Firefox and Internet Explorer 9+. It has a user-friendly visualization interface for easy data uploading and processing, and result interpretation. MAGIC-web is free and open to all users and there is no log in requirement.

## MATERIALS AND METHODS

### Intact glycopeptide MS^2^ spectra processing common in MAGIC and MAGIC+

#### Glycopeptide spectrum filtering

MAGIC can detect B-ions in the low *m/z* region to filter out non-glycopeptide MS^2^ spectra using either specific B-ions, such as the abundant HexNAc+ (*m/z* 204.08) and HexHexNAc+ (*m/z* 366.14) (default setting), or a minimum number (user-specified) of B-ions on the built-in list (Supplementary Table S1).

#### Detecting Y1-ion patterns and generating in silico MS^2^ spectra

The intact glycopeptide MS^2^ spectra are always assigned with the *m/z* of intact glycopeptides as the precursor *m/z* instead of that of the underlying peptides. Directly using such precursor *m/z* to search either conventional protein sequence databases (i.e. untargeted analysis) or user-provided protein sequences (i.e. targeted analysis) will result in false or no identification. Thus, the determination of the underlying peptide precursor *m/z* (i.e. Y0-ion) is critical. Given an intact glycopeptide MS^2^ spectrum, our previously proposed method using two novel triplet peak patterns for Y1-ion detection, called Trident (triplet patterns for accurate Y1-ion identification), is applied to detect two sets of triplet peaks that are matched to consecutive mass losses of either two GlcNAc's (i.e. the [Y0 = peptide, Y1 = Y0 + GlcNAc, Y2 = Y0 + 2GlcNAc] pattern) or the dissociation of an NH_3_ followed by a GlcNAc (i.e. the [Y0-NH_3_, Y0, Y1] pattern) and to determine the Y0-ion (17).

Nonetheless, direct glycopeptide sequence search is not only interfered by the unknown underlying peptide precursor *m/z* but also hampered by B- and Y-ions dominant in both occurrence and intensity in the intact glycopeptide spectra. Therefore, after Y0-ions are detected, dynamic programming is applied on each spectrum to search for other Y-ions as successive peaks above the Y0-ion *m/z* with mass differences that correspond to any monosaccharide. Then, all of the detected B- and Y-ions are removed from the intact glycopeptide spectrum, leaving mostly the b- and y-ions of the underlying peptide, to generate a new *in silico* spectrum, which is further assigned with the Y0-ion *m/z* determined via Trident as the precursor *m/z*. This newly generated set of *in silico* MS^2^ spectra are ready for identifying glycopeptide sequences by directly using a database search engine, e.g. Mascot, against a large-scale public sequence database or using MAGIC+ to search against the user-provided protein sequence file.

#### Glycan composition determination

Finding possible glycan compositions is important in intact glycopeptide analyses. Based on the determined Y0-ion *m/z*, glycan mass can be determined by subtracting Y0-ion *m/z* from the intact glycopeptide *m/z*. Instead of requiring users to upload a glycan database as conventional approaches do, glycan compositions are determined by matching the glycan mass to a look-up table which is constructed to list the exhaustive combinations of up to 29 monosaccharides of various types, with consideration of the general biosynthesis rules for glycan formation. On the basis of detected B- and Y-ions and rules adopted from our previous work, each feasible composition is scored using a new improved scoring function and then ranked.

### Targeted analysis in MAGIC+

For targeted glycopeptide analysis from user-provided protein sequences, MAGIC+ performs the following three steps.

#### Step 1. Generating target-decoy glycopeptide sequences

The input protein sequences are first *in silico* digested by the user-selected enzyme (e.g. trypsin) into peptides so that only peptides containing the sequon (i.e. NXS/T, where X is any amino acid except prolin) are kept. Against this list of potential glycopeptides, *in silico* spectra generated from intact glycopeptide spectra are searched and ranked using the target-decoy approach (18). Compared to a proteomics experiment, the number of identifiable MS^2^ spectra is much less in a glycoproteomics experiment even when a whole proteome is used for evaluation (19). In order to increase the confidence in finding the most plausible glycopeptides that match the generated *in silico* spectra, MAGIC+ generates much more decoy peptide sequences than the target peptide sequences (∼1 target sequence:10 decoy sequences). Specifically, the decoy sequences are generated by (i) reversing the target peptide sequences and (ii) switching the even-odd positioned amino acids (e.g. GLCP will become LGPC) of the target peptide sequences. For each decoy sequence, the sequon is (i) kept at the original location and (ii) randomly inserted in four other locations. However, if a target sequence contains more than one glycosylation sequon, MAGIC+ iterates the process of the random insertion by inserting one of the sequons randomly into other locations while keeping the rest fixed at their original locations. Therefore, each target peptide sequence has ∼10 decoy sequences (repeated sequences will be removed). Here we use the glycoprotein, horseradish peroxidase (HRP), to demonstrate the construction of the customized target-decoy database. Applying *in silico* trypsin digestion (semi-trypsin digestion is not considered), HRP contains seven fully-digested peptides with eight sequons, i.e. LHFHDCFVNGCDASILLD***N***TTSFR, NVGL***N***R, LY***N***FSNTGLPDPTL***N***TTYLQTLR (double sequons), GLCPLNG***N***LSALVDFDLR, GLIQSDQELFSSP***N***ATDTIPLVR, SFA***N***STQTFFNAFVEAMDR and MG***N***ITPLTGTQGQIR. In addition to fully-digested peptides, MAGIC+ considers up to two missed cleavage sites (e.g. DSFRNVGL***N***R has one missed cleavage site in contrast to NVGL***N***R) and possible modifications (carbamidomethylation of cysteine, deamidation of asparagine or glutamine and oxidation of methionine) of each peptide as target peptides. Furthermore, considering detection limitation on MS, each target sequence is restricted to 6–40 amino acids to generate decoy peptide sequences. As a result, the total number of target and decoy peptide sequences for HRP is 429 and 5562, respectively. Then the target and decoy sequences are concatenated to form the customized target-decoy sequence list.

#### Step 2. Glycopeptide sequence determination

Given an *in silico* MS^2^ spectrum, the Y0-ion *m/z* is first searched against the customized target-decoy sequence database. If a match is found (within a user-defined tolerance, 0.6 Da as default setting), the top 40 peaks in the *in silico* MS^2^ spectrum will be compared with the theoretical b- and y-ions of the matched sequence. Particularly when matched to a target sequence, the sequence is considered as a candidate only if at least three b- or y-ions are matched, regardless of the charge states. If only three ions are matched, at least two of the ions have to be consecutive b- or y-ions with charge state of 1+. Note that when a decoy sequence is matched, the number of matched b- or y-ions (with any charge state) could be zero. A peptide score, called PepScore, is calculated based on the intensity ratio between matched b- and y-ions with charge state of 1+ and the top 40 peaks using Equation 1 in the Supplementary File S1.

#### Step 3. Scoring and ranking

**Figure 1.**
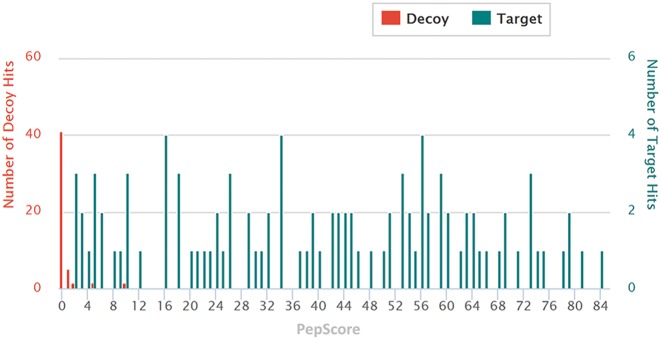
The target-decoy PepScore distribution diagram of the HRP dataset. The y-axis on the left-hand side shows the number of decoy hits and the y-axis on the opposite side shows the number of target hits.

When the search is completed, the candidate sequence (either a target or decoy) with the highest PepScore is considered as the final answer of a given glycopeptide spectrum. In the case where multiple Y1-ion patterns (i.e. multiple Y0-ion *m/z*) are detected in an intact glycopeptide MS^2^ spectrum, each Y0-ion *m/z* is processed to assign a matched peptide sequence. Only the matched sequence and corresponding Y0-ion *m/z* with the most matched b- and y-ions and having a high PepScore are selected to represent the spectrum. Upon completion of processing all of the *in silico* MS^2^ spectra, MAGIC+ applies Mann–Whitney U-test to check whether target hits and decoy hits are closely related or not. A target-decoy score diagram for the entire *in silico* spectra (Figure 1) is provided showing how the scores of the target and decoy hits are distributed.

## RESULTS

### Usage

MAGIC-web provides MAGIC and MAGIC+ for untargeted and targeted glycopeptide analyses, respectively. Reports Integrator combines the results from MAGIC/MAGIC+ and Mascot for more detailed information of identified glycoproteins/glycopeptides. Additionally, Glycan Search module is designed for users with interest in glycans without restricting to N-linked glycosylation. When running any of the modules on the MAGIC-web, the browser does not need to stay open. Users can bookmark the link that is issued after submitting their requests. Alternatively, an email notification with the link will be sent to the user who provides an email address (optional) once the analysis is completed. A user's guide (http://ms.iis.sinica.edu.tw/MAGIC-web/Help.html) is provided with detailed instructions on how to use each of the modules and interpret the results.

### Input

**Figure 2.**
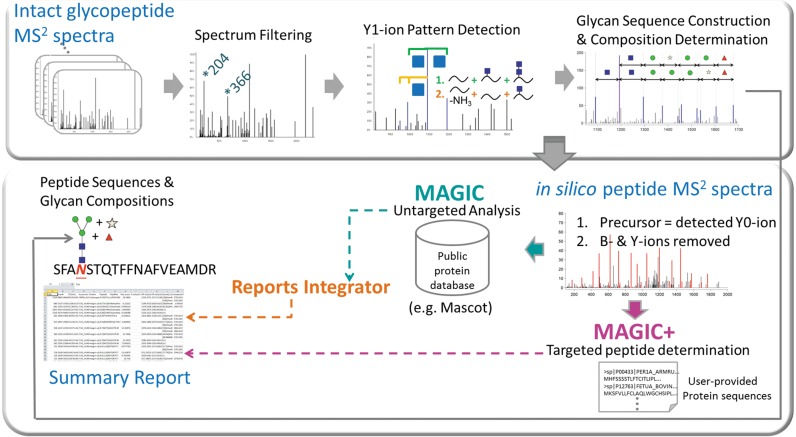
Workflow of MAGIC, MAGIC+ and Reports Integrator for automated untargeted and targeted glycoprotein analyses.

To identify glycopeptides, users need to first click either MAGIC for untargeted analysis or MAGIC+ for targeted analysis on the home page of MAGIC-web. Both MAGIC and MAGIC+ require intact N-linked glycopeptide MS^2^ spectra in Mascot Generic Format (MGF) (Figure 2). For targeted analysis, MAGIC+ additionally requires users to upload their own protein sequences in a text file. Only up to 10 protein sequences are allowed for each targeted analysis. MAGIC and MAGIC+ will generate summary reports after completion of processing the input data.

To combine results from the search engine Mascot for peptide sequencing with the glycopeptide characterization from MAGIC/MAGIC+, users can select Reports Integrator and upload the MGF spectra file, Mascot search result .xml file and .magic file (MAGIC-web configuration file that contains parameter setting and glycan-related information of the input data generated by MAGIC or MAGIC+) to produce a summary report containing proteins and site-specific glycan information.

When using Glycan Search (Supplementary Figure S1) to find interesting glycans, users need to specify the numbers and types of monosaccharides, for instance, a particular glycan composition obtained from MAGIC or MAGIC+, by entering the number in the textbox next to the monosaccharide.

### Output

**Figure 3.**
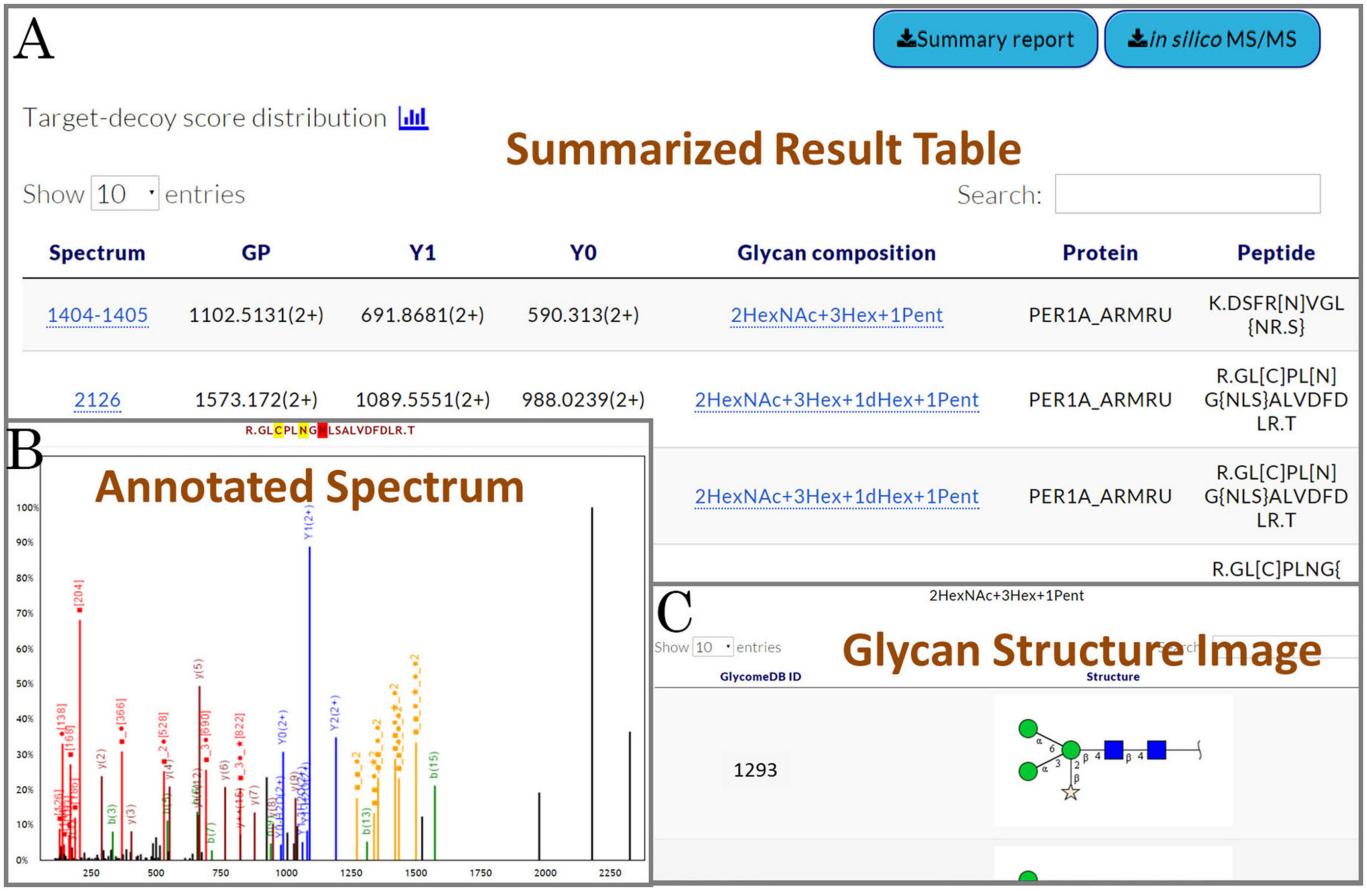
An example of the output with visualization in MAGIC-web. (**A**) The summarized result from MAGIC+ in a simple table. (**B**) Clicking any spectrum number in the ‘Spectrum’ column, its associated annotated spectrum will pop up. Different types of ions are labelled in different colours. (**C**) Images of glycan structures are available for those matched to the calculated glycan compositions.

Upon completion of processing the input data, MAGIC and MAGIC+ will generate a summary report in the interface as a simple table (Figure 3A) to list the spectra that pass the filtering process and contain at least one Y1-ion pattern. A more detailed version of the summarized result is available for download along with the .magic file. Graphical output is provided that an intact glycopeptide spectrum is annotated using different colours to represent different types of ions as shown in Figure 3B (e.g. red is for B-ions whereas blue is for the Y1-ion pattern). Users can click on a glycan composition in blue text to view the possible glycan structures (Figure 3C). MAGIC and MAGIC+ also generate *in silico* MS^2^ peptide spectra for download as well. Please note that MAGIC requires the users to download the *in silico* spectra and use Mascot for identifying glycopeptide sequences, while this is optional for MAGIC+ users since MAGIC+ can automatically assign the glycopeptide sequences.

A summary report containing the search results from Mascot and glycan information from MAGIC (or MAGIC+) can be obtained by using Reports Integrator. Data visualization is available for Reports Integrator as well. Unlike the summary report from MAGIC which lacks protein/peptide information, the summary report obtained from MAGIC+ or Reports Integrator list the identified proteins in alphabetical order. Within each protein, the peptides are alphabetically sorted with consideration of retention time. Score and *E*-value (Mascot only) of each peptide are listed along with their calculated glycan compositions and information of detected Y-ions (e.g. Y0, Y1 and Y2). Images of glycan structures that match user-specified input in Glycan Search are available for users to inspect online. All the information on glycan structures is obtained from GlycomeDB (20).

### MAGIC+ performance

To ensure that MAGIC+ has the ability to accurately determine the possible glycopeptides in the intact glycopeptide MS^2^ spectra, the results from MAGIC+ were compared to our previous findings of two datasets, namely HRP and large-scale HeLa cells (HeLa). These datasets have been used to evaluate stand-alone version of MAGIC. The parameter setting for protein assignment in MAGIC+ was the same as used in Mascot (Matrix Science, London, UK; version 2.4.241) in the previous study. The detailed description of the MS analysis of glycopeptides is in the Supplementary File S1. Further details of the material and reagents, along with the experimental protocols for sample preparation, and settings of data pre-processing and database searching, can be found in the previous publication (17).

The HRP dataset contained total of 734 spectra. Among them, 346 spectra passed the filtering process and contained at least one Y1-ion pattern. MAGIC+ assigned peptide sequences to 100 spectra (Supplementary File S2) using the customized target-decoy database of HRP which contained 429 target sequences and 5562 decoy sequences. MAGIC+ accurately determined the 81 spectra that were identified by Mascot in our previous study (Supplementary Table S2) and assigned glycopeptide sequences to additional 19 spectra.

To further test MAGIC+, instead of using all the 26 glycoprotein sequences that were identified previously in the large-scale HeLa dataset, we randomly selected 5 out of 26 proteins with various length and then *in silico* trypsin-digested into glycopeptides to construct the database with 2547 target sequences and 28425 decoy sequences. The target-decoy approach adopted by MAGIC+ is shown to be effective if MAGIC+ could assign correct glycopeptides in these five selected glycoproteins, bypassing many other spectra corresponding to the glycoproteins absent in the sequence file. As a result, MAGIC+ successfully assigned the 45 previously reported spectra with correct glycopeptide sequences, except one unconfidently identified spectrum of glycoprotein ITA3_HUMAN (Supplementary Table S3 and File S3). This spectrum was not assigned because only two y-ions (y5 and y8) were matched in the spectrum. However, MAGIC+ requires at least three b- or y-ions in order to consider the target peptide sequence as a candidate. Notably, in addition to the glycopeptides that were determined by both Mascot and MAGIC+, five new glycopeptides from four selected glycoproteins, ITA1_HUMAN, ITA3_HUMAN, LAMP1_HUMAN and PCYOX_HUMAN were observed from the dataset. Further examining these glycopeptides in UniProt (http://www.uniprot.org), the glycosylation sites on glycopeptides ***N***MTFDLPSDATVVL***N***R of LAMP1_HUMAN, and MS***N***ITFLNFDPPIEEFHQYYQHIVTTLVK and GEL***N***TSIFSSR of PCYOX_HUMAN have been reported in the literature. The other two glycopeptides, SQNDKF***N***VSLTVK of ITA1_HUMAN and PPGACQA***N***ETIFCELGNPFK of ITA3_HUMAN, are reported by sequence analysis which may be worth further investigation.

## CONCLUSION

Aberrant glycosylation is closely related to many diseases which lead to a pressing need of identifying glycoproteins for discovery of significant biomarkers. However, low abundance of b- and y-ions of underlying peptides in the intact glycopeptide MS^2^ spectra and the challenge of isolating glycopeptides with high purity on the proteome scale, computational methods that can differentiate between glycopeptide and non-glycopeptide spectra and accurately determine site-specific glycopeptides are essential for assisting MS-based glycoproteomics analyses. MAGIC-web offers a user-friendly platform where it can help users to process intact glycopeptide MS^2^ data in batch and deliver accurate results for both untargeted and targeted analyses. MAGIC-web also offers data visualization to support easy result interpretation. The algorithms implemented in MAGIC-web have been extensively tested. To the best of our knowledge, MAGIC-web is the first web-based tool that provides for both untargeted and targeted N-linked glycoprotein analyses.

## Supplementary Material

SUPPLEMENTARY DATA
