# Supplementary material for: MAGIC-web: a platform for untargeted and targeted N-linked glycoprotein identification
Source: Nucleic Acids Res. 2016 Apr 15;44(Web Server issue):W575–80. doi: 10.1093/nar/gkw254 (PMC4987873; doi:10.1093/nar/gkw254)
Supplement: SUPPLEMENTARY DATA [file supp_44_W1_W575__index.html]

MAGIC-web: a platform for untargeted and targeted N-linked glycoprotein identification — MAGIC-web: a platform for untargeted and targeted N-linked glycoprotein identification — SUPPLEMENTARY DATA 

# MAGIC-web: a platform for untargeted and targeted N-linked glycoprotein identification

## SUPPLEMENTARY DATA

- SUPPLEMENTARY DATA
