# Supplementary material for: MAGIC-web: a platform for untargeted and targeted N-linked glycoprotein identification
Source: Nucleic Acids Res. 2016 Apr 15;44(Web Server issue):W575–80. doi: 10.1093/nar/gkw254 (PMC4987873; doi:10.1093/nar/gkw254)
Supplement: SUPPLEMENTARY DATA [file supp_gkw254_nar-00283-web-b-2016-File005.zip › Supplementary File S1.pdf]

MAGIC-web: a platform for untargeted and targeted N-linked glycoprotein identification

T. Mamie Lih[1,2,3], Wai-Kok Choong[2], Chen-Chun Chen[4,5], Cheng-Wei Cheng[1,2,3], Hsin-Nan Lin[2], Ching-Tai Chen[2], Hui-Yin Chang[1,2,3], Wen-Lian Hsu[2] and Ting-Yi Sung[2,*]

[1] Bioinformatics Program, Taiwan International Graduate Program, Academia Sinica, Taipei 11529,Taiwan
[2] Institute of Information Science, Academia Sinica, Taipei 11529, Taiwan
[3] Institute of Biomedical Informatics, National Yang-Ming University, Taipei 11221, Taiwan
[4] Genomics Research Center, Academia Sinica, Taipei 11529, Taiwan
[5] Department of Chemistry, National Taiwan University, Taipei 10617, Taiwan,

Contents

Equation 1 of calculating the PepScore 3
Nano LC-MS^2 analysis 4
Supplementary Table S1. The built-in list of 22 B-ions used in MAGIC and MAGIC+ 5
Supplementary Table S2. Summary of MAGIC+ result comparing to previous finding on the HRP dataset 5
Supplementary Table S3. Summary of MAGIC+ result comparing to previous finding on the HeLa dataset 7
Supplementary Figure S1. An example of how to use Glycan Search and its output. 8

Equation 1 of calculating the PepScore for an *in silico* MS^2 spectrum matches to a target or decoy peptide sequence in MAGIC+.

$$\text{PepScore} = \frac{\sum I_{bi} + \sum I_{yj}}{\sum I_{top40}}$$

where *I* is intensity, *b* and *y* are experimental b- and y-ions with charge state of 1+ from the *in silico* MS^2 spectrum, *i*,*j* = 1,2,…,n (n=peptide length-1), and *top40* is the top 40 peaks (i.e. peaks with high intensities) in the *in silico* MS^2 spectrum.

Nano LC-MS^2 analysis

For LC-MS^2 analysis, the eluate were injected onto a 180 µm × 2 cm capillary trap column and separated on a 75 µm × 25 cm nanoACQUITY®R1.7 µm BEH C18 column. Bound peptides were eluted with a gradient of 0-80% buffer B (0.1% FA in ACN) over 50 and 120 min for standard sample and HeLa cell sample, separately. The sample of single glycoprotein (HRP) was analyzed by a SYNAPT G1 HDMS mass spectrometer (Waters Corp., Manchester, U.K.) under Data Dependent Acquisition (DDA) mode and complex proteomics sample was analyzed by a SYNAPT G2 HDMS mass spectrometer (Waters Corp., Manchester, U.K.) under precursor ion discovery (PID) mode. The MS was operated in ESI positive V mode with a resolving power of 10,000 and calibrated with a synthetic human [Glu1]-Fibrinopeptide B solution (0.5 pmol/µL, Sigma Aldrich, St. Louis, MO, USA) delivered through the NanoLockSpray source. Data acquisition was performed with a full MS scan (*m/z* 400-1600, 0.6 s) followed by three MS^2 (*m/z* 100-1990, 1.2 seconds/scan) sequentially on the three most intense ions present in the full scan mass spectrum. In the PID mode, alternate low (6 eV) and ramping high energy (25 eV to 45 eV) MS survey scans were employed. When *m/z* 204.08 (HexNAc+) and *m/z* 366.13 (HexHexNAc+) were detected at the high-energy survey scans, the three most intense ions observed in the corresponding low-energy survey scans were triggered for their MS^2 acquisition.

Supplementary Table S1. The built-in list of 22 B-ions used in MAGIC and MAGIC+

No.	Marker Type	B-Ion (m/z)
1	Hex[C6H5O2]	109.027
2	Hex[C5H7O3]	115.039
3	HexNAc[C6H8O2N]	126.054
4	Hex-2H2O	127.038976
5	Hex-Several functional groups	138.05
6	HexNAc[C6H10O3N]	144.064
7	Hex[C6H11O5]	145.067
8	Hex	163.060096
9	HexNAc-2H2O	168.065526
10	HexNAc-H2O	186.076086
11	HexNAc	204.086646
12	NeuAc-H_2O	274.0921
13	NeuAc	292.1032
14	Hex+HexNAc	366.139466
15	Hex+NeuAc	454.1561
16	Hex+HexNAc+Fuc	512.1974
17	2Hex+HexNAc	528.192286
18	Hex+HexNAc+NeuAc	657.2354
19	3Hex+HexNAc	690.245106
20	Hex +HexNAc+Fuc +NeuAc	803.2928
21	3Hex+HexNAc+Pent	822.287366
22	3Hex2HexNAc	893.3251

The B-ions listed in the table are reported in the following literature.

1. Ozohanics, O., J. Krenyacz, K. Ludanyi, F. Pollreisz, K. Vekey, L. Drahos (2008). GlycoMiner: a new software tool to elucidate glycopeptide composition. *Rapid Commun. Mass Spectrom.*, **22**, 3245-54.
2. Zhao, P., R. Viner, C. F. Teo, G. J. Boons, D. Horn, L. Wells (2011). Combining high-energy C-trap dissociation and electron transfer dissociation for protein O-GlcNAc modification site assignment. *J. Proteome Res.*, **10**, 4088-104.
3. Balog, C. I. A., O. A. Mayboroda, M. Wuhrer, C. H. Hokke, A. M. Deelder, P. J. Hensbergen (2010). Mass Spectrometric Identification of Aberrantly Glycosylated Human Apolipoprotein C-III Peptides in Urine from Schistosoma mansoni-infected Individuals. *Molecular & Cellular Proteomics*, **9**, 667-681
4. Lin, C. W., J. M. Chen, Y. M. Wang, S. W. Wu, I. H. Tsai, K. H. Khoo (2011). Terminal disialylated multiantennary complex-type N-glycans carried on acutobin define the glycosylation characteristics of the Deinagkistrodon acutus venom. *Glycobiology*, **21**, 530-42

Supplementary Table S2. Summary of MAGIC+ result comparing to previous finding on the HRP dataset

	**Mascot**	**MAGIC+**	
**Peptide Sequence**	Previously reported spectra (confident)	The same spectra as previously reported	Additionally assigned
LHFHDCFVNGCDASILLD^{87}***N***TTSFR	7(7)	7	8
NVGL1^{88}***N***R[#]	7(0)	7	11
LY^{216}***N***FSNTGLPDPTL^{228}***N***TTYLQTLR (double glycosylation sites)	5(3)	5	
GLCPLNG^{244}***N***LSALVDFDLR	9(9)	9	
GLIQSDQELFSSP^{285}***N***ATDTIPLVR	12(8)	12	
SFA^{298}***N***STQTFFNAFVEAMDR	23(23)	23	
MG^{316}**N**ITPLTGTQGQIR	18(11)	18	
**Subtotal**		81	19
**Total**	81(61)	100	

Peptides with missed cleavages were found. The reported number is: K.DSFRNVGL***N***R.S (1 spectrum) and R.NVGL***N***RSSDLVALSGGHTFGKNQCR.F (1 spectrum)

Supplementary Table S3. Summary of MAGIC+ result comparing to previous finding on the HeLa dataset

			**Mascot**	**MAGIC+**	
**Accession**	**Previously Reported Peptide Sequence**	**Newly observed Peptide Sequence**	**Previously Reported Spectra (Conf.)**	**The same spectra as previously reported**	**Additionally assigned**
CBPM	YGEYYLLLLPGSYII^{363}***N***VTVPGHDPHITK		1 (0)	1	
ITA1	^{418}***N***TTFNVESTK		1 (0)	1	
	Y^{460}***N***HTGQVIIYR		4 (2)	4	
		SQNDKF^{699}***N***VSLTVK$^{\%}$			1
ITA3	^{265}***N***ITIVTGAPR		8 (2)	7*	1
	LRPIIISM^{573}***N***YSLPLR		2 (0)	2	3
	KLLLSI^{656}***N***VTNTR$^{\#}$		3 (0)	3	2
		PPGACQA^{697}***N***ETIFCELGNPFK$^{\%}$			1
LAMP1	GHTLTL^{103}***N***FTR		17 (1)	17	4
	LLNINP^{261}***N***KT		6 (0)	6	2
		^{62}***N***MTFDLPSDATVVL^{76}***N***R^{+} (double glycosylation sites)			1
PCYOX	LLHALGGDDFLGML^{196}***N***RT		4 (1)	4	
		MS^{323}***N***ITFLNFDPPIEEFHQYYQHIVTTLVK^{+}			2
		GEL^{353}***N***TSIFSSR^{+}			2
**Subtotal**				45	19
**Total**	9 peptides	5 peptides	46(6)	64	

Peptide with missed cleavages were found. The reported number is: R.KLLLSI***N***VTNTR.T (3 spectrum)

* One spectrum was not assigned due to the matching criteria set in MAGIC+. A spectrum is assigned a target peptide sequence if and only if it contains at least three b- or y-ions. This particular spectrum (unconfidently identified in Mascot) only contained two y-ions (y5 and y8).

+ The glycopeptides with literature confirmations in UniProt.

% These glycopeptides are reported by sequence analysis in UniProt.

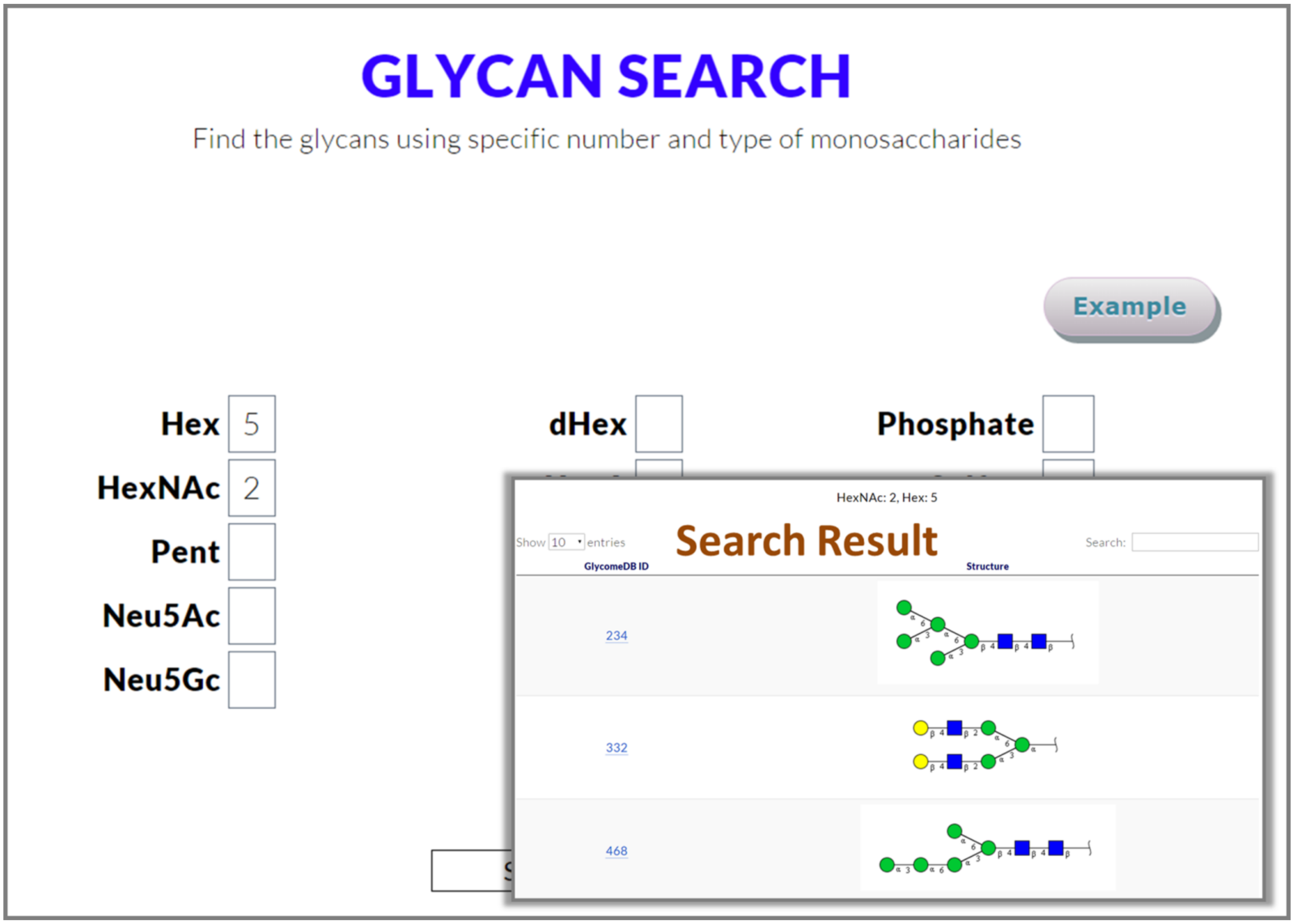

Supplementary Figure S1. An example of how to use Glycan Search and its output. Users need to specify the numbers and types of monosaccharides that they wish to find the associated glycan structures.
